# Efficacy of Xuebijing Injection for Acute Pancreatitis: A Systematic Review and Meta-Analysis of Randomized Controlled Trials

**DOI:** 10.1155/2021/6621368

**Published:** 2021-04-26

**Authors:** Hongxin Chen, Zhaohui Bai, Hongyu Li, Yanyan Wu, Haijuan Yao, Le Wang, Hanyang Lin, Zhenhua Tong, Rolf Teschke, Xingshun Qi

**Affiliations:** ^1^Liver Cirrhosis Study Group, Department of Gastroenterology, General Hospital of Northern Theater Command (formerly General Hospital of Shenyang Military Area), Shenyang, China; ^2^Postgraduate College, Liaoning University of Traditional Chinese Medicine, Shenyang, China; ^3^Department of Life Sciences and Biopharmaceutis, Shenyang Pharmaceutical University, Shenyang, China; ^4^Section of Medical Service, General Hospital of Northern Theater Command (formerly General Hospital of Shenyang Military Area), Shenyang, China; ^5^Department of Internal Medicine II, Division of Gastroenterology and Hepatology, Klinikum Hanau, Academic Teaching Hospital of the Medical Faculty, Goethe University Frankfurt/Main, Frankfurt am Main, D-63450 Hanau, Germany

## Abstract

**Methods:**

PubMed Medline, Embase, Cochrane Library, China National Knowledge Infrastructure, China Biology Medicine disc, VIP, and Wanfang databases were searched. The primary outcome was treatment response. The secondary outcomes included changes in clinical and laboratory indicators and incidence of AP-related complications. Meta-analyses were performed by using a random-effect model. Risk ratios (RRs) with 95% confidence intervals (CIs) or weighted mean differences (WMDs) with 95% CIs were calculated.

**Results:**

Overall, 23 RCTs were included. The rates of overall (RR = 1.16; 95% CI = 1.12 to 1.20; *P* < 0.00001) and complete (RR = 1.40; 95% CI = 1.30 to 1.50; *P* < 0.00001) responses were significantly higher in the Xuebijing injection group. After treatment, the levels of interleukin-6 (WMD = −18.22; 95% CI = −23.36 to −13.08; *P* < 0.00001), tumor necrosis factor-*α* (WMD = −16.44; 95% CI = −20.49 to −12.40; *P* < 0.00001), serum amylase (WMD = −105.61; 95% CI = −173.77 to −37.46; *P*=0.002), white blood cell (WMD = −1.51; 95% CI = −1.66 to −1.36; *P* < 0.00001), and C-reactive protein (WMD = −11.05; 95% CI = −14.32 to −7.78; *P* < 0.00001) were significantly lower in the Xuebijing injection group. Abdominal pain (WMD = −1.74; 95% CI = −1.96 to −1.52; *P* < 0.00001), abdominal distension (WMD = −1.56; 95% CI = −2.07 to −1.04; *P* < 0.00001), gastrointestinal function (WMD = −2.60; 95% CI = −3.07 to −2.13; *P* < 0.00001), body temperature (WMD = −2.16; 95% CI = −2.83 to −1.49; *P* < 0.00001), serum amylase level (WMD = −1.81; 95% CI = −2.66 to −0.96; *P* < 0.0001), and white blood cell (WMD = −2.16; 95% CI = −2.99 to −1.32; *P* < 0.00001) recovered more rapidly in the Xuebijing injection group. The incidence of multiple organ dysfunction syndrome (RR = 0.18; 95% CI = 0.05 to 0.62; *P*=0.006), pancreatic pseudocyst (RR = 0.17; 95% CI = 0.04 to 0.77; *P*=0.02), and renal failure (RR = 0.16; 95% CI = 0.05 to 0.60; *P*=0.006) was significantly lower in the Xuebijing injection group.

**Conclusions:**

Xuebijing injection added on the basis of conventional treatment has a potential benefit for improving the outcomes of AP.

## 1. Introduction

Acute pancreatitis (AP) is one of the most common gastrointestinal diseases that require urgent hospitalization [1, 2]. Its global incidence is 34 cases per 100,000 general population per year [3]. About 20% of AP cases are moderately severe or severe [4]. At present, the overall mortality of AP is about 5% [5], and the mortality of severe AP is up to 30% [6], despite the fact that fluid resuscitation, nutritional support, enzyme suppression, antibiotics, analgesia, and treatment of local and systemic complications have been widely employed [7–10].

Xuebijing injection is a traditional Chinese medicine compound developed by Professor Jinda Wang on the basis of the ancient blood regulating formula and the theory of “simultaneous treatment of bacteria, toxin, and inflammation” [11]. It consists of five Chinese herbs, as follows: Carthami Flos (hong hua), Paeoniae Radix Rubra (chi shao), Chuanxiong Rhizoma (chuan xiong), Salviae Miltiorrhizae Radix et Rhizoma (dan shen), and Angelicae Sinensis Radix (dang gui), which can promote blood circulation, strengthen and consolidate body resistance, clear heat, and remove toxicity [12]. Xuebijing injection has been widely employed for the clinical treatment of AP in China, but its effectiveness still needs to be further confirmed. Additionally, it should be noted that published studies are of poor quality, thereby influencing the reliability of previous findings. Herein, we selected relatively high-quality randomized controlled trials (RCTs) and performed a meta-analysis to clarify the role of Xuebijing injection for the treatment of AP.

## 2. Methods

This meta-analysis was conducted according to the Preferred Reporting Items for Systematic Reviews and Meta-Analyses (PRISMA) statement. The PRIMSA checklist is shown in Supplementary Table 1.

### 2.1. Registration

The meta-analysis was registered in the PROSPERO with a registration number of CRD42020219118.

### 2.2. Search Strategy

PubMed Medline, Embase, Cochrane Library, China National Knowledge Infrastructure (CNKI), China Biology Medicine disc (CBMdisc), VIP, and Wanfang databases were searched. The search items are as follows: “Xuebijing” AND “pancreatitis” (Supplementary Material 1 (available here)). The last search was conducted on November 07, 2020.

### 2.3. Study Selection

There was neither publication language nor publication status restriction. All RCTs regarding Xuebijing injection for the treatment of AP were included. The Xuebijing injection group should include patients, who received Xuebijing injection combined with conventional treatment. The control group should be patients, who received conventional treatment alone.

Exclusion criteria were as follows: (1) duplicates; (2) catalogue, indexes, guidelines, and conferences; (3) reviews or meta-analyses; (4) animal experiments; (5) non-RCTs; (6) irrelevant papers; (7) low-quality RCTs; and (8) absence of efficacy data.

### 2.4. Outcomes of Interest

The primary outcome should be treatment response. The secondary outcomes include laboratory indicators after treatment; recovery time of clinical symptoms and signs and laboratory indicators after treatment; and incidence of AP-related complications.

### 2.5. Data Extraction

The following data were extracted from the included studies: first author; journal; publication year; region; study design; usage and dosage of Xuebijing injection; study population; follow-up duration; classification of AP; laboratory indicators after treatment; recovery time of clinical symptoms and signs and laboratory indicators after treatment; incidence of AP-related complications; and outcomes.

### 2.6. Definitions

The conventional treatment of AP was mainly in accordance with the treatment strategy employed in each included study. Briefly, major treatment strategy included fasting for solids and liquids, fluid resuscitation, gastrointestinal decompression, nutritional support, inhibition of acid and pancreatic secretion, improvement of pancreatic microcirculation, and prophylaxis of infection.

Assessment of response was mainly in accordance with the definitions made by every individual study. In detail, overall response included complete response and partial response; complete response was defined as significant remission or disappearance of clinical symptoms and signs and significant improvement or normalization of laboratory indicators after treatment; partial response was defined as improvement of clinical symptoms and signs and laboratory indicators after treatment; no response was defined as no improvement or even deterioration of clinical symptoms and signs and laboratory indicators after treatment.

### 2.7. Risk of Bias Assessment

The quality of RCTs was assessed using the Cochrane Collaboration's Risk of Bias tool [13], which includes random sequence generation, allocation concealment, blinding of participants and personnel, blinding of outcome assessment, incomplete outcome data, selective reporting, and other bias.

### 2.8. Statistical Analysis

We performed the meta-analysis by the Review Manager 5.2 (Cochrane collaboration, the Nordic Cochrane Centre, Copenhagen, Denmark) and STATA 12.0 (Stata Corp, College Station, Texas, USA). A random-effect model was employed. *P* value < 0.05 was considered statistically significant. Dichotomous outcomes were expressed as risk ratios (RRs) with 95% confidence intervals (CIs), and continuous outcomes were expressed as weighted mean differences (WMDs) with 95% CI. The Cochran's *Q* test and *I*^2^ statistics were employed to assess the heterogeneity. *P* < 0.1 and/or *I*^2^ > 50% was considered as a statistically significant heterogeneity. Subgroup analyses were conducted in patients diagnosed with severe AP. Publication bias was performed with Egger test. *P* < 0.1 was considered as a statistically significant publication bias. The meta-regression analyses and sensitivity analyses were used to explore the sources of heterogeneity. Covariates used for meta-regression analyses included the year of publication, region, and dosage of Xuebijing injection every time. Sensitivity analyses were performed by omitting a single study in turn.

## 3. Results

### 3.1. Study Selection

**Figure 1:**
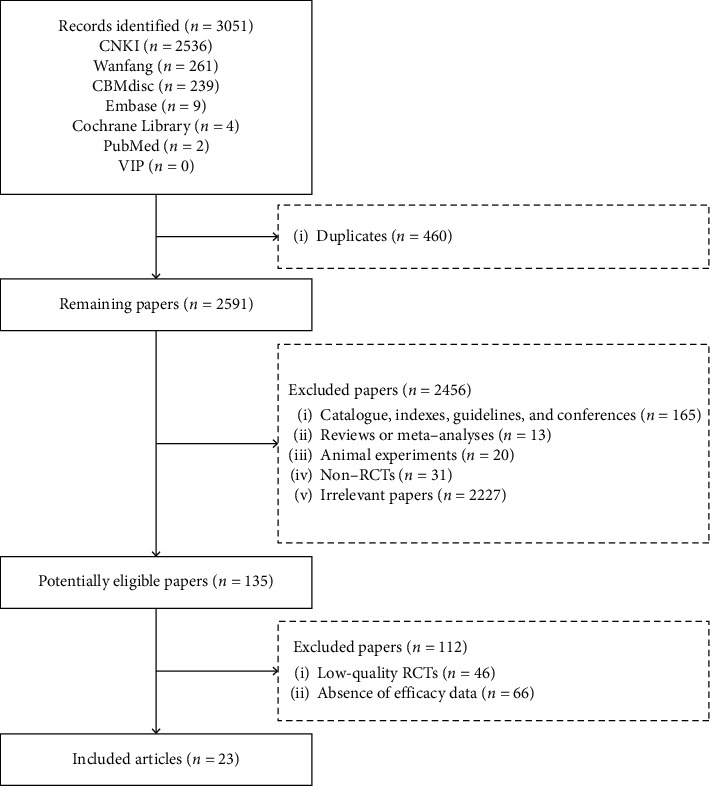
Flow chart of study selection.

Overall, 3051 publications were identified via the 7 databases. Finally, 23 RCTs [14–36] were included in this meta-analysis (Figure 1).

### 3.2. Study Characteristics

The characteristics of these studies are shown in Tables 1 and 2. All of them were conducted in China and published from 2012 to 2020 as full texts. A total of 1882 AP patients were included with a sample size ranging from 39 to 146 among studies.

### 3.3. Risk of Bias

We used the Cochrane Collaboration's Risk of Bias tool to evaluate a total of 69 potentially eligible papers. Among them, 23 RCTs, which had 3 items at a low risk, were included; the remaining 46 papers, which had 2 or even fewer items at a low risk, were excluded (Supplementary Figure 1).

**Figure 2:**
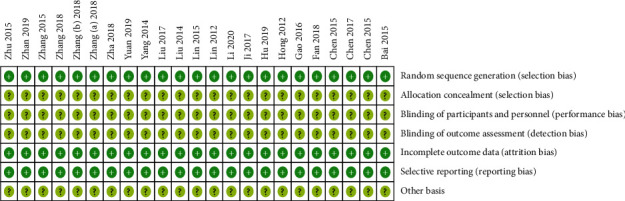
Risk of bias assessment of included RCTs.

In the 23 included RCTs, regarding random sequence generation, all studies had a low risk of bias, of which 20 employed a random number table, 1 employed a computer random number generator, and 2 employed a drawing of lots. Regarding incomplete outcome data and selective reporting, all studies had a low risk of bias. Regarding allocation concealment, blinding of participants and personnel, blinding of outcome assessment, and other bias, all studies had an unclear risk of bias (Figure 2).

### 3.4. Outcomes

#### 3.4.1. Overall Response

**Figure 3:**
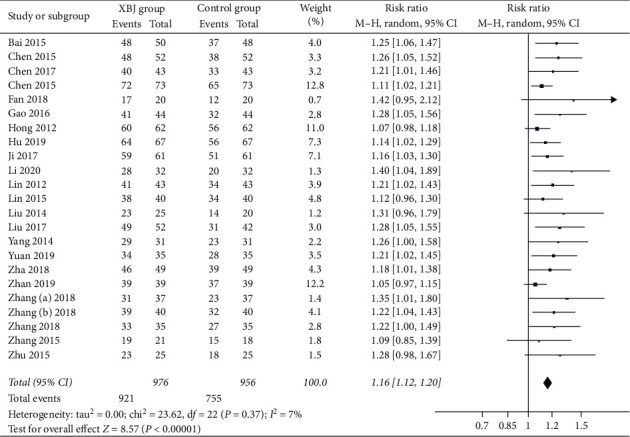
Comparison of overall response between Xuebijing injection and control groups.

Twenty-three studies reported the data regarding overall response. The overall response rate was 94.4% (921/976) in the Xuebijing injection group, and 79.0% (755/956) in the control group. Meta-analysis showed that the Xuebijing injection group had a significantly higher overall response rate than the control group (RR = 1.16; 95% CI = 1.12 to 1.20; *P* < 0.00001) (Figure 3). There was no significant heterogeneity among studies (*I*^2^ = 7%; *P*=0.37).

In the subgroup analysis of severe AP, the Xuebijing injection group also had a significantly higher overall response rate than the control group (RR = 1.17; 95% CI = 1.12 to 1.22; *P* < 0.00001) (Supplementary Figure 2). There was no significant heterogeneity among studies (*I*^2^ = 11%; *P*=0.33).

#### 3.4.2. Complete Response

**Figure 4:**
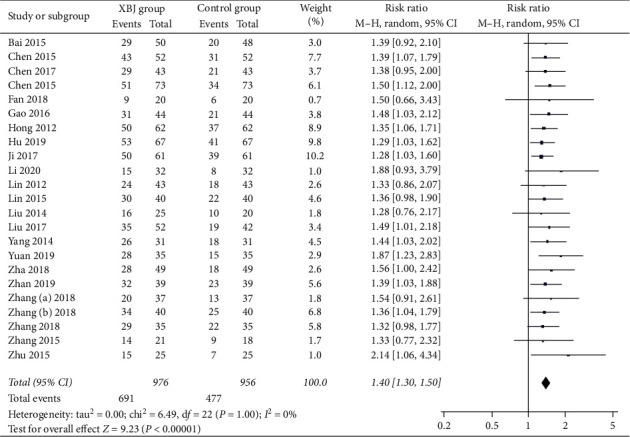
Comparison of complete response between Xuebijing injection and control groups.

Twenty-three studies reported the data regarding complete response. The complete response rate was 70.8% (691/976) in the Xuebijing injection group and 49.9% (477/956) in the control group. Meta-analysis showed that the Xuebijing injection group had a significantly higher complete response rate than the control group (RR = 1.40; 95% CI = 1.30 to 1.50; *P* < 0.00001) (Figure 4). There was no significant heterogeneity among studies (*I*^2^ = 0%; *P*=1.00).

In the subgroup analysis of severe AP, the Xuebijing injection group also had a significantly higher complete response rate than the control group (RR = 1.40; 95% CI = 1.29 to 1.51; *P* < 0.00001) (Supplementary Figure 3). There was no significant heterogeneity among studies (*I*^2^ = 0%; *P*=1.00).

#### 3.4.3. No Response

**Figure 5:**
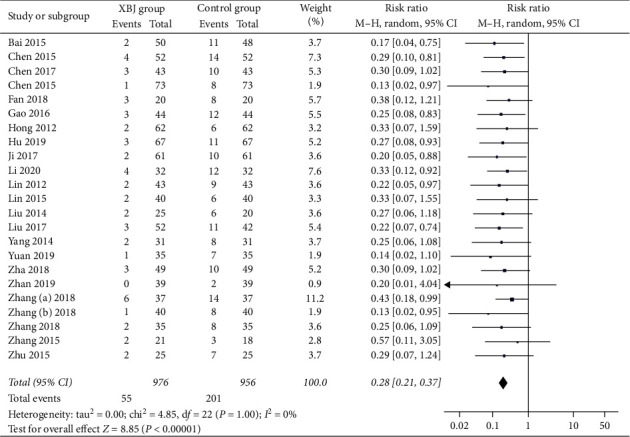
Comparison of no response between Xuebijing injection and control groups.

Twenty-three studies reported the data regarding no response. The non-response rate was 5.6% (55/976) in the Xuebijing injection group and 21.0% (201/956) in the control group. Meta-analysis showed that the Xuebijing injection group had a significantly lower rate of non-response than the control group (RR = 0.28; 95% CI = 0.21 to 0.37; *P* < 0.00001) (Figure 5). There was no significant heterogeneity among studies (*I*^2^ = 0%; *P*=1.00).

In the subgroup analysis of severe AP, the Xuebijing injection group also had a significantly lower rate of non-response than the control group (RR = 0.28; 95% CI = 0.20 to 0.38; *P* < 0.00001) (Supplementary Figure 4). There was no significant heterogeneity among studies (*I*^2^ = 0%; *P*=1.00).

### 3.5. Laboratory Indicators after Treatment

#### 3.5.1. Interleukin-6 Level

Ten studies including 984 patients reported the data regarding interleukin- (IL-) 6 level after treatment. Meta-analysis showed that the Xuebijing injection group had a significantly lower IL-6 level than the control group (WMD = −18.22; 95% CI = −23.36 to −13.08; *P* < 0.00001) (Table 3). There was a significant heterogeneity among studies (*I*^2^ = 97%; *P* < 0.00001). Meta-regression analysis and sensitivity analysis did not find any source of heterogeneity (Supplementary Tables 2 and 3).

#### 3.5.2. Tumor Necrosis Factor-*α* Level

Twelve studies including 1152 patients reported the data regarding tumor necrosis factor-*α* (TNF-*α*) level after treatment. Meta-analysis showed that the Xuebijing injection group had a significantly lower level of TNF-*α* than the control group (WMD = −16.44; 95% CI = −20.49 to −12.40; *P* < 0.00001) (Table 3). There was a significant heterogeneity among studies (*I*^2^ = 97%; *P* < 0.00001). Meta-regression analysis and sensitivity analysis did not find the source of heterogeneity (Supplementary Tables 2 and 3).

#### 3.5.3. Serum Amylase Level

Five studies including 508 patients reported the data regarding serum amylase level after treatment. Meta-analysis showed that the Xuebijing injection group had a significantly lower level of serum amylase than the control group (WMD = −105.61; 95% CI = −173.77 to −37.46; *P*=0.002) (Table 3). There was a significant heterogeneity among studies (*I*^2^ = 95%; *P* < 0.00001). Meta-regression analysis and sensitivity analysis did not find the source of heterogeneity (Supplementary Tables 2 and 3).

#### 3.5.4. White Blood Cell

Six studies including 586 patients reported the data regarding white blood cell (WBC) after treatment. Meta-analysis showed that the Xuebijing injection group had a significantly lower WBC than the control group (WMD = −1.51; 95% CI = −1.66 to −1.36; *P* < 0.00001) (Table 3). There was a significant heterogeneity among studies (*I*^2^ = 88%; *P* < 0.00001). Meta-regression analysis and sensitivity analysis did not find the source of heterogeneity (Supplementary Tables 2 and 3).

#### 3.5.5. C-Reactive Protein Level

Five studies including 560 patients reported the data regarding C-reactive protein level after treatment. Meta-analysis showed that the Xuebijing injection group had a significantly lower level of C-reactive protein value than the control group (WMD = −11.05; 95% CI = −14.32 to −7.78; *P* < 0.00001) (Table 3). There was a significant heterogeneity among studies (*I*^2^ = 95%; *P* < 0.00001). Meta-regression analysis and sensitivity analysis did not find the source of heterogeneity (Supplementary Tables 2 and 3).

#### 3.5.6. Hypersensitive C-Reactive Protein Level

Four studies including 380 patients reported the data regarding hypersensitive C-reactive protein level after treatment. Meta-analysis showed that the Xuebijing injection group had a significantly lower level of hypersensitive C-reactive protein than the control group (WMD = −12.39; 95% CI = −19.34 to −5.44; *P*=0.0005) (Table 3). There was a significant heterogeneity among studies (*I*^2^ = 96%; *P* < 0.00001). Meta-regression analysis and sensitivity analysis did not find the source of heterogeneity (Supplementary Tables 2 and 3).

### 3.6. Recovery Time of Clinical Symptoms and Signs and Laboratory Indicators after Treatment

#### 3.6.1. Abdominal Pain

Twelve studies including 1093 patients reported the recovery time of abdominal pain. Meta-analysis showed that the Xuebijing injection group had a significantly shorter recovery time of abdominal pain than the control group (WMD = −1.74; 95% CI = −1.96 to −1.52; *P* < 0.00001) (Table 4). There was a significant heterogeneity among studies (*I*^2^ = 41%; *P*=0.07). Meta-regression analysis did not find the source of heterogeneity (Supplementary Table 2). Sensitivity analysis found that the heterogeneity became not significant after excluding the study by Lin et al. (*I*^2^ = 37%; *P*=0.10) and Zhang et al. (*I*^2^ = 19%; *P*=0.26) (Supplementary Table 3).

#### 3.6.2. Abdominal Distension

Seven studies including 637 patients reported the recovery time of abdominal distension. Meta-analysis showed that the Xuebijing injection group had a significantly shorter recovery time of abdominal distension than the control group (WMD = −1.56; 95% CI = −2.07 to −1.04; *P* < 0.00001) (Table 4). There was a significant heterogeneity among studies (*I*^2^ = 79%; *P* < 0.0001). Meta-regression analysis and sensitivity analysis did not find the source of heterogeneity (Supplementary Tables 2 and 3).

#### 3.6.3. Gastrointestinal Function

Six studies including 536 patients reported the recovery time of gastrointestinal function. Meta-analysis showed that the Xuebijing injection group had a significantly shorter recovery time of gastrointestinal function than the control group (WMD = −2.60; 95% CI = −3.07 to −2.13; *P* < 0.00001) (Table 4). There was a significant heterogeneity among studies (*I*^2^ = 89%; *P* < 0.00001). Meta-regression analysis and sensitivity analysis did not find the source of heterogeneity (Supplementary Tables 2 and 3).

#### 3.6.4. Body Temperature

Six studies including 551 patients reported the recovery time of body temperature. Meta-analysis showed that the Xuebijing injection group had a significantly shorter recovery time of body temperature than the control group (WMD = −2.16; 95% CI = −2.83 to −1.49; *P* < 0.00001) (Table 4). There was a significant heterogeneity among studies (*I*^2^ = 85%; *P* < 0.00001). Meta-regression analysis and sensitivity analysis did not find the source of heterogeneity (Supplementary Tables 2 and 3).

#### 3.6.5. Serum Amylase Level

Five studies including 385 patients reported the recovery time of serum amylase level. Meta-analysis showed that the Xuebijing injection group had a significantly shorter recovery time of serum amylase level than the control group (WMD = −1.81; 95% CI = −2.66 to −0.96; *P* < 0.0001) (Table 4). There was a significant heterogeneity among studies (*I*^2^ = 84%; *P* < 0.0001). Meta-regression analysis and sensitivity analysis did not find the source of heterogeneity (Supplementary Tables 2 and 3).

#### 3.6.6. WBC

Eight studies including 631 patients reported the recovery time of WBC. Meta-analysis showed that the Xuebijing injection group had a significantly shorter recovery time of WBC than the control group (WMD = −2.16; 95% CI = −2.99 to −1.32; *P* < 0.00001) (Table 4). There was a significant heterogeneity among studies (*I*^2^ = 86%; *P* < 0.00001). Meta-regression analysis and sensitivity analysis did not find the source of heterogeneity (Supplementary Tables 2 and 3).

### 3.7. AP-Related Complications

#### 3.7.1. Multiple Organ Dysfunction Syndrome

Four studies including 368 patients reported the incidence of multiple organ dysfunction syndrome. Meta-analysis showed that the Xuebijing injection group had a significantly lower incidence of multiple organ dysfunction syndrome than the control group (RR = 0.18; 95% CI = 0.05 to 0.62; *P*=0.006) (Table 5). There was no significant heterogeneity among studies (*I*^2^ = 0%; *P*=0.94).

#### 3.7.2. Acute Respiratory Distress Syndrome

Three studies including 193 patients reported the incidence of acute respiratory distress syndrome. Meta-analysis showed that the Xuebijing injection group had a lower incidence of acute respiratory distress syndrome than the control group, but there was no significant difference between the two groups (RR = 0.38; 95% CI = 0.14 to 1.04; *P*=0.06) (Table 5). There was no significant heterogeneity among studies (*I*^2^ = 0%; *P*=0.55).

#### 3.7.3. Septicemia

Three studies including 294 patients reported the incidence of septicemia. Meta-analysis showed that the Xuebijing injection group had a lower incidence of septicemia than the control group, but there was no significant difference between the two groups (RR = 0.67; 95% CI = 0.19 to 2.36; *P*=0.54) (Table 5). There was no significant heterogeneity among studies (*I*^2^ = 0%; *P*=0.54).

#### 3.7.4. Pancreatic Pseudocyst

Three studies including 265 patients reported the incidence of pancreatic pseudocyst. Meta-analysis showed that the Xuebijing injection group had a significantly lower incidence of pancreatic pseudocyst than the control group (RR = 0.17; 95% CI = 0.04 to 0.77; *P*=0.02) (Table 5). There was no significant heterogeneity among studies (*I*^2^ = 0%; *P*=0.80).

#### 3.7.5. Shock

Two studies including 123 patients reported the incidence of shock. Meta-analysis showed that the Xuebijing injection group had a lower incidence of shock than the control group, but there was no significant difference between the two groups (RR = 0.30; 95% CI = 0.08 to1.12; *P*=0.07) (Table 5). There was no significant heterogeneity among studies (*I*^2^ = 23%; *P*=0.25).

#### 3.7.6. Renal Failure

Two studies including 152 patients reported the incidence of renal failure. Meta-analysis showed that the Xuebijing injection group had a significantly lower incidence of renal failure than the control group (RR = 0.16; 95% CI = 0.05 to 0.60; *P*=0.006) (Table 5). There was no significant heterogeneity among studies (*I*^2^ = 0%; *P*=0.55).

#### 3.7.7. Pleural Effusion or Ascites

Two studies including 119 patients reported the incidence of pleural effusion or ascites. Meta-analysis showed that the Xuebijing injection group had a lower incidence of pleural effusion or ascites than the control group, but there was no significant difference between the two groups (RR = 0.35; 95% CI = 0.12 to 1.01; *P*=0.05) (Table 5). There was no significant heterogeneity among studies (*I*^2^ = 6%; *P*=0.30).

### 3.8. Safety

In our included studies, none reported the data regarding adverse events related to Xuebijing injection.

### 3.9. Publication Bias

Publication bias is reported in Supplementary Table 4.

## 4. Discussion

Our systematic review suggests that Xuebijing injection combined with conventional treatment is more effective for AP than conventional treatment alone. Similar findings could be observed in the subgroup analysis of severe AP. Our study has several major features in study design and statistical analysis. First, we conducted more extensive literature search and included a larger number of publications. Second, we excluded low-quality RCTs. Third, we explored the efficacy outcomes in more details, including overall response, complete response, and no response. Fourth, we explored the laboratory indicators after treatment between Xuebijing injection and control groups. Fifth, we analyzed the incidence of various complications of AP between the two groups. Sixth, we conducted the meta-regression analyses to explore the source of heterogeneity.

In AP, the initial mediator that induces inflammatory cell response is TNF-*α*, which is the earliest promoter of inflammatory mediator chain reaction [37]. TNF-*α* induces the expression of many inflammatory factors, such as IL-6 [38], which react on macrophages, thereby producing more TNF-*α* [39]. This vicious cycle triggers the inflammatory cascade reaction, causing toxic damage to pancreas and other organs [40, 41].

It has been confirmed that Xuebijing injection can improve microcirculation, increase blood flow, reduce inflammation and capillary permeability, decrease inflammatory exudation, promote inflammation absorption, and inhibit the formation of inflammatory granulomas, which can alleviate the pathological damage during the infection [42]. Xuebijing injection is the only patented Chinese medicine officially authorized for the treatment of systemic inflammatory response syndrome and multiple organ dysfunction syndrome [43]. In the current clinical practice, Xuebijing injection has been widely used to treat critical diseases, such as sepsis [12], acute respiratory distress syndrome [44], severe pneumonia [45], and spontaneous bacterial peritonitis [46]. Xuebijing injection has a strong ability to antagonize endotoxin, which can effectively block the uncontrolled release of endogenous inflammatory mediators produced by endotoxin-induced monocytes/macrophages, thereby preventing from inflammation reaction [42, 46]. In addition, it can effectively reduce the levels of TNF-*α*, hypersensitive C-reactive protein, and IL-6, which are pro-inflammatory factors, upregulate the IL-10 level, which is an anti-inflammatory factor, block the cascade reaction mediated by inflammatory factors, and finally reduce the inflammatory reaction [11]. Our meta-analysis showed that Xuebijing injection can significantly reduce the levels of inflammatory mediators.

The incidence of adverse reactions of Xuebijing injection is about 0.3%, which often develops within 30 minutes of medication [47]. Most of them are mild. Adverse reactions are mainly located at the respiratory system, skin, and accessories. Main manifestations are skin pruritus, erythema, and chest tightness [48]. Based on the findings of the present systematic review, no adverse reactions related to Xuebijing injection have been reported.

Our meta-analysis has several major limitations. First, the treatment strategy of the control group was not completely equal. Second, the follow-up duration was too short. The average duration is always 7 days, and the longest duration is only 14 days. Third, blinding and allocation concealment were not reported in all included studies. Fourth, most of the included studies had a small sample size and were conducted at a single center. Fifth, all of included studies were conducted in China.

In conclusion, the application of Xuebijing injection on the basis of conventional treatment can improve the outcomes of AP. However, Xuebijing injection is currently used in China alone. In the future, more high-quality, well-designed, multi-center, and large-scale RCTs are still needed to validate the clinical efficacy and safety of Xuebijing injection for the treatment of AP.

## Figures and Tables

**Table 1:** Characteristics of included studies.

First author (year)	Region	Journal	Study design	Classification of AP	Groups	Interventions	Duration of treatment (days)
Li (2020)	Shanxi	Guangming Journal of Chinese Medicine	RCT	SAP	XBJ group	Conventional treatment; Xuebijing injection 100 ml, 2 time/day	14
					Control group	Conventional treatment	
Zhan (2019)	Beijing	Chinese Archives of Traditional Chinese Medicine	RCT	SAP	XBJ group	Conventional treatment; Xuebijing injection 100 ml, 2 times/day	10
					Control group	Conventional treatment	
Yuan (2019)	Hubei	Chinese and Foreign Medical Research	RCT	SAP	XBJ group	Conventional treatment; Xuebijing injection 100 ml, 2 times/day	7
					Control group	Conventional treatment	
Hu (2019)	Zhejiang	Chinese Journal of Surgery of Integrated Traditional and Western Medicine	RCT	SAP	XBJ group	Conventional treatment; Xuebijing injection 100 ml, 1 time/day	10
					Control group	Conventional treatment	
Zhang (2018)	Henan	Chinese Journal of New Clinical Medicine	RCT	SAP	XBJ group	Conventional treatment; Xuebijing injection 100 ml, 2 times/day	7
					Control group	Conventional treatment	
Zhang (a) (2018)	Shaanxi	Modern Journal of Integrated Traditional Chinese and Western Medicine	RCT	SAP	XBJ group	Conventional treatment; Xuebijing injection 100 ml, 2 times/day	7
					Control group	Conventional treatment	
Zhang (b) (2018)	Shaanxi	Medical Journal of West China	RCT	SAP	XBJ group	Conventional treatment; Xuebijing injection 50 ml, 2 times/day	7
					Control group	Conventional treatment	
Fan (2018)	Chongqing	Journal of Clinical Medical	RCT	SAP	XBJ group	Conventional treatment; Xuebijing injection 100 ml, 2 times/day	7
					Control group	Conventional treatment	
Zha (2018)	Henan	Chongqing Medicine	RCT	SAP	XBJ group	Conventional treatment; Xuebijing injection 100 ml, 2 times/day	7
					Control group	Conventional treatment	
Ji (2017)	Qinghai	Shaanxi Journal of Traditional Chinese Medicine	RCT	SAP	XBJ group	Conventional treatment; Xuebijing injection 50 ml, 2–3 times/day	7
					Control group	Conventional treatment	
Chen (2017)	Henan	Modern Diagnosis and Treatment	RCT	SAP	XBJ group	Conventional treatment; Xuebijing injection 100 ml, 2 times/day	7
					Control group	Conventional treatment	
Liu (2017)	Hubei	World Chinese Journal of Digestology	RCT	SAP	XBJ group	Conventional treatment; Xuebijing injection 100 ml, 2 times/day	7
					Control group	Conventional treatment	
Gao (2016)	Liaoning	Chinese Traditional Patent Medicine	RCT	SAP	XBJ group	Conventional treatment; Xuebijing injection 100 ml, 2 times/day	7
					Control group	Conventional treatment	
Chen (2015)	Hubei	World Chinese Journal of Digestology	RCT	SAP	XBJ group	Conventional treatment; Xuebijing injection 100 ml, 2 times/day	7
					Control group	Conventional treatment	
Chen (2015)	Hebei	International Journal of Traditional Chinese Medicine	RCT	SAP	XBJ group	Conventional treatment; Xuebijing injection 50 ml, 2 times/day	7
					Control group	Conventional treatment	
Zhang (2015)	Liaoning	Medical Information	RCT	AP	XBJ group	Conventional treatment; Xuebijing injection 50 ml, 2 times/day	NA
					Control group	Conventional treatment	
Zhu (2015)	Jiangsu	The World Clinical Medicine	RCT	AP	XBJ group	Conventional treatment; Xuebijing injection 50 ml, 2 times/day	NA
					Control group	Conventional treatment	
Liu (2015)	Zhejiang	Journal of Emergency in Traditional Chinese Medicine	RCT	AP	XBJ group	Conventional treatment; Xuebijing injection 100 ml, 2 times/day	7
					Control group	Conventional treatment	
Bai (2015)	Nei Mongol	China Foreign Medical Treatment	RCT	AP	XBJ group	Conventional treatment; Xuebijing injection 50 ml, 2 times/day	7
					Control group	Conventional treatment	
Yang (2014)	Fujian	Fujian Journal of Traditional Chinese Medicine	RCT	AP	XBJ group	Conventional treatment; Xuebijing injection 50 ml, 2 times/day	5–7
					Control group	Conventional treatment	
Liu (2014)	Henan	Modern Journal of Integrated Traditional Chinese and Western Medicine	RCT	SAP	XBJ group	Conventional treatment; Xuebijing injection 50 ml, 2 times/day	7
					Control group	Conventional treatment	
Lin (2012)	Xinjiang	Nei Mongol Journal of Traditional Chinese Medicine	RCT	SAP	XBJ group	Conventional treatment; Xuebijing injection 100 ml, 2 times/day	7
					Control group	Conventional treatment	
Hong (2012)	Zhejiang	Journal of Emergency in Traditional Chinese Medicine	RCT	AP	XBJ group	Conventional treatment; Xuebijing injection 100 ml, 2 times/day	7
					Control group	Conventional treatment	

Abbreviations: RCT: randomized controlled trial; AP: acute pancreatitis; SAP: severe acute pancreatitis; XBJ: Xuebijing injection.

**Table 2:** Characteristics of included patients.

First author (year)	No. pts	Etiology of acute pancreatitis	Sex (male/female) (*n*)	Age (mean)	Groups	Follow-up (days)	Overall response (*n*)	Complete response (*n*)	No response (*n*)
Li (2020)	32	NA	16/16	43.1	XBJ group	14	28	15	4
	32	NA	17/15	43.6	Control group	14	20	8	12
Zhan (2019)	39	Biliary diseases 14; alcohol 11; surgery 4; overeating 10	28/11	46.6	XBJ group	10	39	32	0
	39	Biliary diseases 15; alcohol 11; surgery 5; overeating 7; other 1	24/15	45.9	Control group	10	37	23	2
Yuan (2019)	35	Biliary diseases 13; alcohol 15; hyperlipidemia 7	19/16	50.7	XBJ group	7	34	28	1
	35	Biliary diseases 13; alcohol 13; hyperlipidemia 9	21/14	49.8	Control group	7	28	15	7
Hu (2019)	67	NA	40/27	41.5	XBJ group	10	64	53	3
	67	NA	39/28	41.4	Control group	10	56	41	11
Zhang (2018)	35	NA	19/16	42.8	XBJ group	7	33	29	2
	35	NA	20/15	43.1	Control group	7	27	22	8
Zhang (a) (2018)	37	NA	18/19	39.3	XBJ group	7	31	20	6
	37	NA	17/20	38.0	Control group	7	23	13	14
Zhang (b) (2018)	40	Biliary diseases 14; alcohol 11; surgery 4; overeating 10	23/17	42.1	XBJ group	7	39	34	1
	40	Biliary diseases 20; alcohol 10; overeating 9; other 1	25/15	40.5	Control group	7	32	25	8
Fan (2018)	20	NA	11/9	38.6	XBJ group	7	17	9	3
	20	NA	12/8	38.9	Control group	7	12	6	8
Zha (2018)	49	NA	NA	NA	XBJ group	7	46	28	3
	49	NA	NA	NA	Control group	7	39	18	10
Ji (2017)	61	NA	48/13	48.0	XBJ group	7	59	50	2
	61	NA	46/15	48.2	Control group	7	51	39	10
Chen (2017)	43	NA	24/19	43.1	XBJ group	7	40	29	3
	43	NA	25/18	43.3	Control group	7	33	21	10
Liu (2017)	52	NA	29/23	42.5	XBJ group	7	49	35	3
	42	NA	24/18	40.5	Control group	7	31	19	11
Gao (2016)	44	NA	23/21	41.9	XBJ group	7	41	31	3
	44	NA	24/20	42.6	Control group	7	32	21	12
Chen (2015)	73	Biliary diseases 43; alcohol or overeating 30	45/28	42.7	XBJ group	7	72	51	1
	73	Biliary diseases 42; alcohol or overeating 31	46/27	42.8	Control group	7	65	34	8
Chen (2015)	52	Biliary diseases 25; alcohol 109; overeating 8; hyperlipidemia 6; other 3	30/22	53.6	XBJ group	7	48	43	4
	52	Biliary diseases 24; alcohol 9; overeating 9; hyperlipidemia 8; other 2	31/21	53.7	Control group	7	38	31	14
Zhang (2015)	21	NA	NA	NA	XBJ group	NA	19	14	2
	18	NA	NA	NA	Control group	NA	15	9	3
Zhu (2015)	25	NA	17/8	36.5	XBJ group	NA	23	15	2
	25	NA	18/7	36.7	Control group	NA	18	7	7
Liu (2015)	40	NA	28/12	32.6	XBJ group	7	38	30	2
	40	NA	26/14	30.4	Control group	7	34	22	6
Bai (2015)	50	NA	33/17	46.7	XBJ group	7	48	29	2
	48	NA	30/18	49.0	Control group	7	37	20	11
Yang (2014)	31	NA	22/9	40.3	XBJ group	5–7	29	26	2
	31	NA	20/11	41.2	Control group	5–7	23	18	8
Liu (2014)	25	Biliary diseases 2; alcohol 12; overeating 9; other 2	16/9	48.2	XBJ group	7	23	16	2
	20	Biliary diseases 4; alcohol 9; overeating 5; other 2	13/7	49.1	Control group	7	14	10	6
Lin (2012)	43	NA	26/17	38.8	XBJ group	7	41	24	2
	43	NA	28/15	39.2	Control group	7	34	18	9
Hong (2012)	62	NA	40/22	44.5	XBJ group	7	60	50	2
	62	NA	40/22	44.9	Control group	7	56	37	6

Abbreviations: Pts: patients; XBJ: Xuebijing injection.

**Table 3:** Meta-analyses of laboratory indicators after treatment.

Endpoints	No. studies	Pooled proportion using random-effects model		Heterogeneity	
		WMD	*P*	*I* ^2^ (%)	*P*
IL-6 level	10	−18.22 (95% CI = −23.36 to −13.08)	<0.00001	97	<0.00001
TNF-*α* level	12	−16.44 (95% CI = −20.49 to −12.40)	<0.00001	97	<0.00001
AMS level	5	−105.61 (95% CI = −173.77 to −37.46)	0.002	95	<0.00001
WBC	6	−1.51 (95% CI = −1.66 to −1.36)	<0.00001	88	<0.00001
CRP level	5	−11.05 (95% CI = −14.32 to −7.78)	<0.00001	95	<0.00001
hs-CRP level	4	−12.39 (95% CI = −19.34 to −5.44)	0.0005	96	<0.00001

Abbreviations: WMD: weighted mean difference; CI: confidence Interval; IL-6: interleukin-6; TNF-*α*: tumor necrosis factor-*α*; AMS: serum amylase; WBC: white blood cell; CRP: C-reactive protein; hs-CRP: hypersensitive C-reactive protein.

**Table 4:** Meta-analyses of recovery time clinical symptoms and signs and laboratory indicators after treatment.

Endpoints	No. of studies	Pooled proportion using random-effects model		Heterogeneity	
		WMD	*P*	*I* ^2^ (%)	*P*
Abdominal pain	12	−1.74 (95% CI = −1.96 to −1.52)	<0.00001	41	0.07
Abdominal distension	7	−1.56 (95% CI = −2.07 to −1.04)	<0.00001	79	<0.0001
Gastrointestinal function	6	−2.60 (95% CI = −3.07 to −2.13)	<0.00001	89	<0.00001
Body temperature	6	−2.16 (95% CI = −2.83 to −1.49)	<0.00001	85	<0.00001
AMS level	5	−1.81 (95% CI = −2.66 to −0.96)	<0.0001	84	<0.0001
WBC	8	−2.16 (95% CI = −2.99 to −1.32)	<0.00001	86	<0.00001

Abbreviations: WMD: weighted mean difference; CI: confidence Interval; AMS: serum amylase; WBC: white blood cell.

**Table 5:** Meta-analyses of AP-related complications.

Endpoints	No. of studies	Pooled proportion using random-effects model		Heterogeneity	
		RR	*P*	*I* ^2^ (%)	*P*
MODS	4	0.18 (95% CI = 0.05 to 0.62)	0.006	0	0.94
ARDS	3	0.38 (95% CI = 0.14 to 1.04)	0.06	0	0.55
Septicemia	3	0.67 (95% CI = 0.19 to 2.36)	0.54	0	0.54
Pancreatic pseudocyst	3	0.17 (95% CI = 0.04 to 0.77)	0.02	0	0.80
Shock	2	0.30 (95% CI = 0.08 to 1.12)	0.07	23	0.25
Renal failure	2	0.16 (95% CI = 0.05 to 0.60)	0.006	0	0.55
Pleural effusion or ascites	2	0.35 (95% CI = 0.12 to 1.01)	0.05	6	0.30

Abbreviations: AP: acute pancreatitis; RR: risk ratio; CI: confidence Interval; MODS: multiple organ dysfunction syndrome; ARDS: acute respiratory distress syndrome.

## Abbreviations

AP: Acute pancreatitis
CBMdisc: China Biology Medicine disc
CNKI: China National Knowledge Infrastructure
CIs: Confidence intervals
IL: Interleukin
RRs: Risk ratios
RCTs: Randomized controlled trials
TNF-*α*: Tumor necrosis factor-*α*
WBC: White blood cell
WMDs: Weighted mean differences.

## Supplementary Materials

Supplementary Materials Supplementary Material 1: search strategy. Supplementary Material 2: Supplementary Figure 1: risk of bias assessment of potentially eligible papers. Supplementary Material 3: Supplementary Figure 2: comparison of overall response between Xuebijing injection and control groups in subgroup analyses of severe acute pancreatitis. Supplementary Material 4: Supplementary Figure 3: comparison of complete response between Xuebijing injection and control groups in subgroup analyses of severe acute pancreatitis. Supplementary Material 5: Supplementary Figure 4: comparison of no response between Xuebijing injection and control groups in subgroup analyses of severe acute pancreatitis. Supplementary Material 6: Supplementary Table 1: the Preferred Reporting Items for Systematic Reviews and Meta-Analyses (PRISMA) checklist. Supplementary Material 7: Supplementary Table 2: meta-regression analyses. Supplementary Material 8: Supplementary Table 3: sensitivity analyses. Supplementary Material 9: Supplementary Table 4: publication bias.

## References

[1] Tenner S., Baillie J., DeWitt J., Vege S. S. (2013). American college of gastroenterology guideline: management of acute pancreatitis. *American Journal of Gastroenterology*.

[2] Peery A. F., Dellon E. S., Lund J. (2012). Burden of gastrointestinal disease in the United States: 2012 update. *Gastroenterology*.

[3] Petrov M. S., Yadav D. (2019). Global epidemiology and holistic prevention of pancreatitis. *Nature Reviews Gastroenterology and Hepatology*.

[4] van Dijk S. M., Hallensleben N. D. L., van Santvoort H. C. (2017). Acute pancreatitis: recent advances through randomised trials. *Gut*.

[5] Du Y., Chen Q., Yu H. (2019). Chinese guidelines for the management of acute pancreatitis (Shenyang, 2019). *Journal of Clinical Hepatology*.

[6] Waller A., Long B., Koyfman A., Gottlieb M. (2018). Acute pancreatitis: updates for emergency clinicians. *The Journal of Emergency Medicine*.

[7] Lankisch P. G., Apte M., Banks P. A. (2015). Acute pancreatitis. *The Lancet*.

[8] Lee P. J., Papachristou G. I. (2019). New insights into acute pancreatitis. *Nature Reviews Gastroenterology and Hepatology*.

[9] Majidi S., Golembioski A., Wilson S. L., Thompson E. C. (2017). Acute pancreatitis: etiology, pathology, diagnosis, and treatment. *Southern Medical Journal*.

[10] Greenberg J. A., Hsu J., Bawazeer M. (2016). Clinical practice guideline: management of acute pancreatitis. *Canadian Journal of Surgery*.

[11] Teng Y., Sun F., Zhang W. (2015). Research Progress on the mechanism of Xuebijing injection in the treatment of acute pancreatitis. *Chinese Journal of Integrated Traditional and Western Medicine in Intensive and Critical Care*.

[12] Li C., Wang P., Zhang L. (2018). Efficacy and safety of Xuebijing injection (a Chinese patent) for sepsis: a meta-analysis of randomized controlled trials. *Journal of Ethnopharmacology*.

[13] Higgins J. P. T., Altman D. G., Gotzsche P. C. (2011). The cochrane collaboration’s tool for assessing risk of bias in randomised trials. *BMJ*.

[14] Yuan B. (2019). Clinical value of Xuebijing injection in adjuvant treatment of severe acute pancreatitis. *Chinese and Foreign Medical Research*.

[15] Bai Y., Liang Y. (2015). Clinical analysis of Xuebijing in the treatment of acute pancreatitis. *China Foreign Medical Treatment*.

[16] Chen C., Xie P. (2015). Clinical study on Xuebijing injection combined with conventional western medicine therapy in the treatment of severe acute pancreatitis. *International Journal of Traditional Chinese Medicine*.

[17] Chen L., Zhang Y. (2017). Clinical observation of Xuebijing combined with somatostatin in the treatment of severe acute pancreatitis. *Modern Diagnosis and Treatment*.

[18] Chen Q., Yin H. (2015). Clinical effects of anisodamine combined with Xuebijing in treatment of severe pancreatitis. *World Chinese Journal of Digestology*.

[19] Gao P., Hu Z. (2016). Clinical efficacy of Xuebijing combined with somatostatin in treatment of severe acute pancreatitis. *Chinese Traditional Patent Medicine*.

[20] Hong L., Zhan Y., Pan X. (2012). Clinical observation of Xuebijing injection in the treatment of acute pancreatitis and its effect on serum high sensitivity C-reactive protein. *Journal of Emergency in Traditional Chinese Medicine*.

[21] Hu Y., Shan Y., Huang P. (2019). Effect of ulinastatin combined with xuebijing on severe acute pancreatitis. *Chinese Journal of Surgery of Integrated Traditional and Western Medicine*.

[22] Zhang J. (2018). Effect of Xuebijing combined with ulinastatin in the treatment of severe acute pancreatitis and its influence on serum inflammatory factors. *Chinese Journal of New Clinical Medicine*.

[23] Ji H., Yang P. (2017). Effect of Xuebijing injection combined with ulinastatin on serum inflammatory factors and clinical efficacy in patients with severe acute pancreatitis. *Shaanxi Journal of Traditional Chinese Medicine*.

[24] Zhang L. (2015). Analysis of Xuebijing in the treatment of acute pancreatitis. *Medical Information*.

[25] Zhu L. (2015). Clinical effect of somatostatin and pantoprazole combined with Xuebijing in the treatment of acute pancreatitis. *The World Clinical Medicine*.

[26] Li G., Lv Y. (2020). Effect of Xuebijing Injection on Hemorheology and TNF-*α* and IL-6 levels in patients with severe acute pancreatitis. *Guangming Journal of Chinese Medicine*.

[27] Lin F., Lin Y., Hong J. (2012). Clinical observation of Xuebijing injection combined with ulinastatin in the treatment of severe acute pancreatitis. *Nei Mongol Journal of Traditional Chinese Medicine*.

[28] Liu S., Li Z. (2015). Clinical observation of Xuebijing injection combined with western medicine in the treatment of acute pancreatitis. *Journal of Emergency in Traditional Chinese Medicine*.

[29] Liu W. (2014). Effect of Xuebijing Injection on plasma endothelin and ammonia monoxide levels in patients with severe pancreatitis. *Modern Journal of Integrated Traditional Chinese and Western Medicine*.

[30] Dong X. (2017). Xuebijing Injection for treatment of severe acute pancreatitis: curative effect and influence on inflammatory factors. *World Chinese Journal of Digestology*.

[31] Fan Y. (2018). Clinical observation of Xuebijing combined with esomeprazole in the treatment of severe acute pancreatitis. *Journal of Clinical Medical*.

[32] Yang J., Zhang R., Chen H. (2014). Xuebijing injection treated 31 cases of acute pancreatitis. *Fujian Journal of Traditional Chinese Medicine*.

[33] Zha L., Gu Y., Zhao M. (2018). Clinical observation of ulinastatin combined with Xuebijing in the treatment of severe acute pancreatitis. *Chongqing Medicine*.

[34] Zhan Y., Xu F., Fang Z. (2019). Effect of Xuebijing injection combined with octreotide and ulinastatin in treatment of severe acute pancreatitis and its influence on immune function. *Chinese Archives of Traditional Chinese Medicine*.

[35] Zhang H., Wang C., Zhang Y. (2018). Clinical effecacy of stilamin combined with Xuebijing in the treatment of severe acute pancreatitis and its effect on serum IL-10, IL-18 and TNF-*α*. *Medical Journal of West China*.

[36] Zhang H., Zhang N., Niu D. (2018). Clinical observation of Xuebijing combined with esomeprazole in the treatment of severe acute pancreatitis. *Modern Journal of Integrated Traditional Chinese and Western Medicine*.

[37] Guo J., Wang Y. (2013). The effection of Xuebijing injection on serum TNF-*α* IL-6 and IL-10 in severe acute pancreatitis. *China Modern Doctor*.

[38] Sandoval J., Pereda J., Rodriguez J. L. (2010). Ordered transcriptional factor recruitment and epigenetic regulation of tnf-*α* in necrotizing acute pancreatitis. *Cellular and Molecular Life Sciences*.

[39] Pereda J., Sabater L., Aparisi L. (2006). Interaction between cytokines and oxidative stress in acute pancreatitis. *Current Medicinal Chemistry*.

[40] Zeng J., Chen N. (2013). The regulation of immune cytokines by Xuebijing combined with ulinastatin in treatment of severe acute pancreatitis. *Practical Journal of Clinical Medicine*.

[41] Xiao H., Tang K. (2016). Effects of Xuebijing injection combined with ulinastain on endotoxin and inflammatory factors in patients with severe acute pancreatitis. *Journal of Hainan Medical University*.

[42] Zhang W., Li Z., Wang J. (2006). Clinical observation of Xubijing injection in treating 42 cases of severe acute pancreatitis. *Chinese Journal of Critical Care Medicine*.

[43] Li C., Wang P., Li M. (2020). The current evidence for the treatment of sepsis with Xuebijing injection: bioactive constituents, findings of clinical studies and potential mechanisms. *Journal of Ethnopharmacology*.

[44] Dai L., Chen M., Zhuang Y. (2019). Meta-analysis of the clinical efficacy of xuebijing injection in the treatment of acute respiratory distress syndrome. *World Latest Medicine Information*.

[45] Han D., Wang R., Yu Y. (2018). Xuebijing injection combined with antibiotics for the treatment of spontaneous bacterial peritonitis in liver cirrhosis: a meta-analysis. *Evidence-Based Complementary and Alternative Medicine*.

[46] Song Y., Yao C., Yao Y. (2019). Xuebijing injection versus placebo for critically ill patients with severe community-acquired pneumonia. *Critical Care Medicine*.

[47] Nie A., Guo Z., Zhang Y. (2019). Literature analysis of 211 cases of adverse reactions of Xuebijing injection. *Strait Pharmaceutical Journal*.

[48] Zheng R., Wang H., Liu Z. (2019). A real-world study on adverse drug reactions to Xuebijing injection: hospital intensive monitoring based on 93 hospitals (31,913 cases). *Annals of Translational Medicine*.

