# Supplementary material for: Efficacy of Xuebijing Injection for Acute Pancreatitis: A Systematic Review and Meta-Analysis of Randomized Controlled Trials
Source: Evid Based Complement Alternat Med. 2021 Apr 26;2021:6621368. doi: 10.1155/2021/6621368 (PMC8214658; doi:10.1155/2021/6621368)
Supplement: Supplementary Materials — Supplementary Material 1: search strategy. Supplementary Material 2: Supplementary Figure 1: risk of bias assessment of potentially eligible papers. Supplementary Material 3: Supplementary Figure 2: comparison of overall response between Xuebijing injection and control groups in subgroup analyses of severe acute pancreatitis. Supplementary Material 4: Supplementary Figure 3: comparison of complete response between Xuebijing injection and control groups in subgroup analyses of severe acute pancreatitis. Supplementary Material 5: Supplementary Figure 4: comparison of no response between Xuebijing injection and control groups in subgroup analyses of severe acute pancreatitis. Supplementary Material 6: Supplementary Table 1: the Preferred Reporting Items for Systematic Reviews and Meta-Analyses (PRISMA) checklist. Supplementary Material 7: Supplementary Table 2: meta-regression analyses. Supplementary Material 8: Supplementary Table 3: sensitivity analyses. Supplementary Material 9: Supplementary Table 4: publication bias. [file 6621368.f1.zip › 6621368.f1/Supplementary Figure 1.pdf]

	Random sequence generation (selection bias)	Allocation concealment (selection bias)	Blinding of participants and personnel (performance bias)	Blinding of outcome assessment (detection bias)	Incomplete outcome data (attrition bias)	Selective reporting (reporting bias)	Other bias
Bai Y 2015	+	?	?	?	+	+	?
Chen C 2015	+	?	?	?	+	+	?
Cheng Y 2014	?	?	?	?	+	+	?
Chen L 2017	+	?	?	?	+	+	?
Chen Q 2015	+	?	?	?	+	+	?
Cui J 2011	?	?	?	?	+	+	?
Du J 2016	?	?	?	?	+	+	?
Fan Y 2018	+	?	?	?	+	+	?
Fu S 2009	?	?	?	?	+	+	?
Fu Z 2011	?	?	?	?	+	+	?
Gao P 2016	+	?	?	?	+	+	?
Geng D 2012	?	?	?	?	+	+	?
Guo J 2013	?	?	?	?	+	+	?
He Q 2008	?	?	?	?	+	+	?
Hong L 2012	+	?	?	?	+	+	?
Huang P 2016	+	?	?	?	+	+	−
Hu G 2010	?	?	?	?	+	+	?
Hu Y 2019	+	?	?	?	+	+	?
Ji H 2017	+	?	?	?	+	+	?
Ji W 2014	?	?	?	?	+	+	?
Kuang C 2017	?	?	?	?	+	+	?
Kui Y 2015	?	?	?	?	+	+	?
Leng K 2008	?	?	?	?	+	−	?
Liang Y 2017	?	?	?	?	+	+	?
Li G 2018	?	?	?	?	+	+	?
Li G 2020	+	?	?	?	+	+	?
Lin F 2012	+	?	?	?	+	+	?
Ling Z 2014	?	?	?	?	+	+	?
Lin Z 2011	?	?	?	?	+	+	?
Liu D 2009	?	?	?	?	+	+	?
Liu S 2015	+	?	?	?	+	+	?
Liu W 2014	+	?	?	?	+	+	?
Liu X 2017	+	?	?	?	+	+	?
Lu X 2014	?	?	?	?	+	+	?
Ma Q 2016	?	?	?	?	+	+	?
Miu L 2019	?	?	?	?	+	+	?
Qi Q 2017	?	?	?	?	−	+	?
Ruan M 2011	?	?	?	?	+	+	?
Shen H 2009	?	?	?	?	+	+	?
Tian X 2015	?	?	?	?	+	+	?
Wang Z 2011	?	?	?	?	+	+	?
Wei L 2016	?	?	?	?	+	+	?
Wei W 2015	?	?	?	?	+	+	?
Wu H 2012	?	?	?	?	+	+	?
Wu H 2013	?	?	?	?	+	+	?
Wu W 2009	?	?	?	?	−	+	?
Xu Z 2011	?	?	?	?	+	+	?
Yang J 2014	+	?	?	?	+	+	?
Yang Y 2011	?	?	?	?	+	+	?
Ye G 2012	?	?	?	?	+	+	?
Yuan B 2019	+	?	?	?	+	+	?
Yue Y 2014	?	?	?	?	+	+	?
Yue Y 2016	?	?	?	?	+	+	?
Zha L 2018	+	?	?	?	+	+	?
Zhang H(a) 2018	+	?	?	?	+	+	?
Zhang H(b) 2018	+	?	?	?	+	+	?
Zhang J 2018	+	?	?	?	+	+	?
Zhang L 2015	+	?	?	?	+	+	?
Zhang W 2006	?	?	?	?	+	+	?
Zhang W 2010	?	?	?	?	−	+	?
Zhang W 2018	+	?	?	?	?	+	−
Zhang X 2009	?	?	?	?	+	+	?
Zhang X 2016	?	?	?	?	+	+	?
Zhan Y 2019	+	?	?	?	+	+	?
Zhao J 2010	?	?	?	?	+	+	?
Zhou H 2014	?	?	?	?	+	+	?
Zhou Y 2015	?	?	?	?	+	+	?
Zhu K 2013	?	?	?	?	+	+	?
Zhu L 2015	+	?	?	?	+	+	?
