# Supplementary material for: Efficacy of Xuebijing Injection for Acute Pancreatitis: A Systematic Review and Meta-Analysis of Randomized Controlled Trials
Source: Evid Based Complement Alternat Med. 2021 Apr 26;2021:6621368. doi: 10.1155/2021/6621368 (PMC8214658; doi:10.1155/2021/6621368)
Supplement: Supplementary Materials — Supplementary Material 1: search strategy. Supplementary Material 2: Supplementary Figure 1: risk of bias assessment of potentially eligible papers. Supplementary Material 3: Supplementary Figure 2: comparison of overall response between Xuebijing injection and control groups in subgroup analyses of severe acute pancreatitis. Supplementary Material 4: Supplementary Figure 3: comparison of complete response between Xuebijing injection and control groups in subgroup analyses of severe acute pancreatitis. Supplementary Material 5: Supplementary Figure 4: comparison of no response between Xuebijing injection and control groups in subgroup analyses of severe acute pancreatitis. Supplementary Material 6: Supplementary Table 1: the Preferred Reporting Items for Systematic Reviews and Meta-Analyses (PRISMA) checklist. Supplementary Material 7: Supplementary Table 2: meta-regression analyses. Supplementary Material 8: Supplementary Table 3: sensitivity analyses. Supplementary Material 9: Supplementary Table 4: publication bias. [file 6621368.f1.zip › 6621368.f1/Supplementary Table 2 (2).docx]

| **Supplementary Table 2. Meta-regression analyses.** | | | | | | |
| --- | --- | --- | --- | --- | --- | --- |
| **Endpoints** | **Coefficient** | **Standard error.** | **t** | **P** | **95% Confidence interval** | |
| ***IL-6 level*** | | | | | | |
| Year | -0.1083 | 2.8008 | -0.04 | 0.970 | -6.9617 | 6.7451 |
| Region | -1.5636 | 4.8201 | -0.32 | 0.757 | -13.3581 | 10.2309 |
| Dosage | 0.4020 | 7.8644 | 0.05 | 0.961 | -18.8415 | 19.6454 |
| ***TNF-α level*** | | | | | | |
| Year | 0.5331 | 1.4153 | 0.38 | 0.716 | -2.7307 | 3.7968 |
| Region | -1.4311 | 5.9319 | -0.24 | 0.815 | -15.1101 | 12.2478 |
| Dosage | 1.6860 | 4.9700 | 0.34 | 0.743 | -9.7749 | 13.1468 |
| ***AMS level*** | | | | | | |
| Year | 43.6490 | 72.5953 | 0.60 | 0.655 | -878.7613 | 966.0593 |
| Region | -3.5500 | 95.9339 | -0.04 | 0.976 | -1222.5060 | 1215.4060 |
| Dosage | -19.4370 | 91.1068 | -0.21 | 0.866 | -1177.0590 | 1138.1850 |
| ***WBC*** | | | | | | |
| Year | 0.4293 | 0.8032 | 0.53 | 0.646 | -3.0264 | 3.8850 |
| Region | -1.8191 | 2.6492 | -0.69 | 0.563 | -13.2179 | 9.5796 |
| Dosage | 1.3393 | 1.8562 | 0.72 | 0.546 | -6.6472 | 9.3258 |
| ***CRP level*** | | | | | | |
| Year | -0.0050 | 1.7280 | 0.00 | 0.998 | -21.9616 | 21.9516 |
| Region | -1.3700 | 6.9180 | -0.20 | 0.876 | -89.2722 | 86.5322 |
| Dosage | 1.3399 | 5.7256 | 0.23 | 0.854 | -71.4106 | 74.0903 |
| ***hs-CRP level*** | | | | | | |
| Region | 5.9827 | 6.0369 | 0.99 | 0.426 | -19.9922 | 31.9576 |
| ***Recovery time of abdominal pain*** | | | | | | |
| Year | -0.0553 | 0.2324 | -0.24 | 0.817 | -0.5810 | 0.4704 |
| Region | -0.0367 | 1.3659 | -0.03 | 0.979 | -3.1266 | 3.0531 |
| Dosage | 0.3311 | 0.8463 | 0.39 | 0.705 | -1.5835 | 2.2456 |
| ***Recovery time of abdominal distension*** | | | | | | |
| Year | -0.0482 | 0.3974 | -0.12 | 0.911 | -1.3128 | 1.2163 |
| Region | 0.0477 | 1.9168 | 0.02 | 0.982 | -6.0524 | 6.1477 |
| Dosage | 0.2405 | 2.0692 | 0.12 | 0.915 | -6.3447 | 6.8256 |
| ***Recovery time of gastrointestinal function*** | | | | | | |
| Year | -0.0162 | 0.5837 | -0.03 | 0.980 | -2.5279 | 2.4954 |
| Region | -0.3389 | 1.7173 | -0.20 | 0.862 | -7.7278 | 7.0499 |
| Dosage | 0.0072 | 1.0300 | 0.01 | 0.995 | -4.4244 | 4.4388 |
| ***Recovery time of body temperature*** | | | | | | |
| Year | 0.1953 | 0.4884 | 0.40 | 0.728 | -1.9063 | 2.2969 |
| Region | -0.6738 | 2.8733 | -0.23 | 0.836 | -13.0368 | 11.6892 |
| Dosage | 0.8281 | 1.5866 | 0.52 | 0.654 | -5.9985 | 7.6546 |
| ***Recovery time of AMS level*** | | | | | | |
| Year | 0.0983 | 0.5545 | 0.18 | 0.888 | -6.9479 | 7.1445 |
| Region | 0.2457 | 1.8475 | 0.13 | 0.916 | -23.2291 | 23.7206 |
| Dosage | 0.2974 | 3.0753 | 0.10 | 0.939 | -38.7776 | 39.3724 |
| ***Recovery time of WBC*** | | | | | | |
| Year | -0.1303 | 0.6402 | -0.20 | 0.849 | -1.9078 | 1.6471 |
| Region | 0.3446 | 1.8992 | 0.18 | 0.865 | -4.9285 | 5.6177 |
| Dosage | -0.3466 | 2.9465 | -0.12 | 0.912 | -8.5273 | 7.8341 |
| **Abbreviations:** IL-6: interleukin-6; TNF-α: tumor necrosis factor-α; AMS: serum amylase; WBC: white blood cell; CRP: C-reactive protein; hs-CRP: high sensitivity C-reactive protein. | | | | | | |
