# Supplementary material for: Efficacy of Xuebijing Injection for Acute Pancreatitis: A Systematic Review and Meta-Analysis of Randomized Controlled Trials
Source: Evid Based Complement Alternat Med. 2021 Apr 26;2021:6621368. doi: 10.1155/2021/6621368 (PMC8214658; doi:10.1155/2021/6621368)
Supplement: Supplementary Materials — Supplementary Material 1: search strategy. Supplementary Material 2: Supplementary Figure 1: risk of bias assessment of potentially eligible papers. Supplementary Material 3: Supplementary Figure 2: comparison of overall response between Xuebijing injection and control groups in subgroup analyses of severe acute pancreatitis. Supplementary Material 4: Supplementary Figure 3: comparison of complete response between Xuebijing injection and control groups in subgroup analyses of severe acute pancreatitis. Supplementary Material 5: Supplementary Figure 4: comparison of no response between Xuebijing injection and control groups in subgroup analyses of severe acute pancreatitis. Supplementary Material 6: Supplementary Table 1: the Preferred Reporting Items for Systematic Reviews and Meta-Analyses (PRISMA) checklist. Supplementary Material 7: Supplementary Table 2: meta-regression analyses. Supplementary Material 8: Supplementary Table 3: sensitivity analyses. Supplementary Material 9: Supplementary Table 4: publication bias. [file 6621368.f1.zip › 6621368.f1/Supplementary Table 3 (3).docx]

| **Supplementary table 3. Sensitivity analyses** | | |
| --- | --- | --- |
| **Study omitted** | **WMD (95%CI)** | **I^2^ ; P value** |
| ***laboratory indicators after treatment.*** | |  |
| **IL-6 level** |  |  |
| Chen C (2015) | -16.90 (-21.99, -11.80) | 97%; P<0.00001 |
| Chen Q (2015) | -15.66 (-20.22, -11.10) | 95%; P<0.00001 |
| Hu Y (2019) | -20.13 (-25.96, -14.30) | 97%; P<0.00001 |
| Ji H (2017) | -18.87 (-24.66, -13.08) | 97%; P<0.00001 |
| Li G (2020) | -16.03 (-21.07, -10.98) | 97%; P<0.00001 |
| Liu X (2017) | -20.02 (-25.68, -14.36) | 97%; P<0.00001 |
| Zha L (2018) | -20.15 (-25.97, -14.33) | 97%; P<0.00001 |
| Zhan Y (2019) | -19.72 (-25.76, -13.68) | 97%; P<0.00001 |
| Zhang H(a) (2018) | -18.92 (-24.64, -13.21) | 97%; P<0.00001 |
| Zhang H(b) (2018) | -16.36 (-21.43, -11.28) | 97%; P<0.00001 |
| **TNF-α level** |  |  |
| Chen C (2015) | -17.43 (-21.70, -13.16) | 97%; P<0.00001 |
| Chen Q (2015) | -17.47 (-22.41, -12.53) | 97%; P<0.00001 |
| Gao P (2016) | -17.01 (-21.32, -12.70) | 97%; P<0.00001 |
| Hu Y (2019) | -17.54 (-22.02, -13.06) | 97%; P<0.00001 |
| Ji H (2017) | -17.44 (-22.13, -12.74) | 97%; P<0.00001 |
| Li G (2020) | -14.83 (-18.84, -10.82) | 97%; P<0.00001 |
| Liu X (2017) | -17.12 (-21.50, -12.74) | 97%; P<0.00001 |
| Zha L (2018) | -17.52 (-21.65, -13.40) | 96%; P<0.00001 |
| Zhan Y (2019) | -16.27 (-20.51, -12.02) | 97%; P<0.00001 |
| Zhang H(a) (2018) | -17.44 (-21.72, -13.15) | 97%; P<0.00001 |
| Zhang H(b) (2018) | -16.63 (-20.90, -12.37) | 97%; P<0.00001 |
| Zhang J (2018) | -12.15 (-15.22, -9.09) | 94%; P<0.00001 |
| **AMS level** |  |  |
| Bai Y (2015) | -121.69 (-208.96, -34.42) | 95%; P<0.00001 |
| Chen C (2015) | -107.18 (-187.05, -27.32) | 96%; P<0.00001 |
| Chen Q (2015) | -109.23 (-218.66, 0.20) | 96%; P<0.00001 |
| Liu S (2015) | -71.16 (-101.26, -41.07) | 63%; P<0.00001 |
| Zhang J (2018) | -115.68 (-192.22, -39.13) | 96%; P<0.00001 |
| **WBC** |  |  |
| Bai Y (2015) | -1.56 (-1.71, -1.41) | 86%; P<0.0001 |
| Chen C (2015) | -1.48 (-1.63, -1.33) | 88%; P<0.00001 |
| Chen Q (2015) | -2.06 (-2.56, -1.56) | 89%; P<0.00001 |
| Liu S (2015) | -1.48 (-1.63, -1.33) | 87%; P<0.00001 |
| Zhan Y (2019) | -1.49 (-1.64, -1.34) | 87%; P<0.00001 |
| Zhang J (2018) | -1.51 (-1.66, -1.36) | 90%; P<0.00001 |
| **CRP level** |  |  |
| Bai Y (2015) | -12.13 (-15.44, -8.81) | 90%; P<0.00001 |
| Chen C (2015) | -11.83 (-15.50, -8.16) | 96%; P<0.00001 |
| Chen Q (2015) | -10.84 (-15.57, -6.11) | 96%; P<0.00001 |
| Hu Y (2019) | -11.10 (-15.62, -6.59) | 96%; P<0.00001 |
| Zhan Y (2019) | -9.40 (-11.79, -7.02) | 89%; P<0.00001 |
| **hs-CRP level** |  |  |
| Gao P (2016) | -11.66 (-21.10, -2.22) | 97%; P<0.00001 |
| Hong L (2012) | -14.29 (-22.51, -6.07) | 94%; P<0.00001 |
| Liu X (2017) | -9.26 (-14.40, -4.12) | 91%; P<0.0001 |
| Zhang H(a) (2018) | -14.42 (-23.30, -5.54) | 97%; P<0.00001 |
| ***Recovery time clinical symptoms and signs and laboratory indicators after treatment.*** | | |
| **Abdominal pain** |  |  |
| Bai Y (2015) | -1.71 (-1.93, -1.48) | 43%; P=0.06 |
| Chen C (2015) | -1.76 (-1.98, -1.54) | 41%; P=0.08 |
| Chen L (2017) | -1.77 (-2.00, -1.55) | 39%; P=0.09 |
| Chen Q (2015) | -1.70 (-1.96, -1.44) | 46%; P=0.05 |
| Gao P (2016) | -1.77 (-2.00, -1.55) | 39%; P=0.09 |
| Ji H (2017) | -1.69 (-1.93, -1.46) | 41%; P=0.08 |
| Lin F (2012) | -1.78 (-1.99, -1.56) | ***37%; P=0.10*** |
| Liu S (2015) | -1.72 (-1.95, -1.48) | 46%; P=0.05 |
| Liu W (2014) | -1.75 (-1.98, -1.52) | 44%; P=0.06 |
| Zha L (2018) | -1.75 (-1.98, -1.51) | 45%; P=0.05 |
| Zhan Y (2019) | -1.76 (-1.99, -1.53) | 43%; P=0.06 |
| Zhang J (2018) | -1.68 (-1.88, -1.49) | ***19%; P=0.26*** |
| **Abdominal distension** | |  |
| Chen C (2015) | -1.69 (-2.20, -1.17) | 79%; P=0.0009 |
| Chen Q (2015) | -1.39 (-1.88, -0.89) | 67%; P=0.010 |
| Lin F (2012) | -1.60 (-2.16, -1.04) | 81%; P<0.0001 |
| Liu S (2015) | -1.54 (-2.15, -0.92) | 82%; P<0.0001 |
| Liu W (2014) | -1.59 (-2.16, -1.02) | 81%; P<0.0001 |
| Zha L (2018) | -1.67 (-2.20, -1.15) | 76%; P=0.0008 |
| Zhan Y (2019) | -1.42 (-2.04, -0.80) | 80%; P=0. 0001 |
| **Gastrointestinal function** | |  |
| Chen C (2015) | -2.46 (-2.72, -2.20) | 55%; P=0.07 |
| Chen L (2017) | -2.59 (-3.17, -2.00) | 91%; P<0.00001 |
| Gao P (2016) | -2.58 (-3.17, -1.99) | 91%; P<0.00001 |
| Yang J (2014) | -2.78 (-3.22, -2.33) | 89%; P<0.00001 |
| Zhang H(a) (2018) | -2.61 (-3.19, -2.04) | 91%; P<0.00001 |
| Zhang J (2018) | -2.58 (-3.13, -2.03) | 91%; P<0.00001 |
| **Body temperature** |  |  |
| Chen C (2015) | -2.35 (-3.11, -1.59) | 86%; P<0.0001 |
| Chen Q (2015) | -2.24 (-3.22, -1.27) | 87%; P<0.00001 |
| Liu S (2015) | -2.07 (-2.81, -1.33) | 87%; P<0.00001 |
| Liu W (2014) | -2.34 (-3.11, -1.57) | 86%; P<0.00001 |
| Zha L (2018) | -1.83 (-2.28, -1.38) | 59%; P=0.04 |
| Zhan Y (2019) | -2.12 (-2.91, -1.33) | 87%; P<0.00001 |
| **AMS level** |  |  |
| Ji H (2017) | -1.51 (-2.28, -0.73) | 74%; P=0.009 |
| Lin F (2012) | -2.03 (-2.96, -1.09) | 84%; P=0.0003 |
| Liu W (2014) | -1.85 (-2.90, -0.80) | 88%; P<0.0001 |
| Yang J (2014) | -2.55 (-3.31, -1.79) | 82%; P=0.0009 |
| Yuan B (2019) | -1.62 (-2.63, -0.61) | 86%; P=0.0001 |
| **WBC** |  |  |
| Bai Y (2015) | -2.42 (-2.92, -1.92) | 46%; P=0.09 |
| Ji H (2017) | -2.14 (-3.10, -1.18) | 88%; P<0.00001 |
| Lin F (2012) | -2.23 (-3.15, -1.31) | 88%; P<0.00001 |
| Liu W (2014) | -2.17 (-3.12, -1.23) | 88%; P<0.00001 |
| Yang J (2014) | -2.15 (-3.12, -1.19) | 88%; P<0.00001 |
| Yuan B (2019) | -2.21 (-3.16, -1.27) | 88%; P<0.00001 |
| Zha L (2018) | -2.03 (-2.92, -1.15) | 84%; P<0.00001 |
| Zhu L (2015) | -1.91 (-2.73, -1.10) | 85%; P<0.00001 |

**Abbreviations:** WMD: Weighted Mean Difference; CI: Confidence Interval; IL-6: interleukin-6; TNF-α: tumor necrosis factor-α; AMS: serum amylase; WBC: white blood cell; CRP: C-reactive protein; hs-CRP: high sensitivity C-reactive protein.
