# Supplementary material for: Efficacy of Xuebijing Injection for Acute Pancreatitis: A Systematic Review and Meta-Analysis of Randomized Controlled Trials
Source: Evid Based Complement Alternat Med. 2021 Apr 26;2021:6621368. doi: 10.1155/2021/6621368 (PMC8214658; doi:10.1155/2021/6621368)
Supplement: Supplementary Materials — Supplementary Material 1: search strategy. Supplementary Material 2: Supplementary Figure 1: risk of bias assessment of potentially eligible papers. Supplementary Material 3: Supplementary Figure 2: comparison of overall response between Xuebijing injection and control groups in subgroup analyses of severe acute pancreatitis. Supplementary Material 4: Supplementary Figure 3: comparison of complete response between Xuebijing injection and control groups in subgroup analyses of severe acute pancreatitis. Supplementary Material 5: Supplementary Figure 4: comparison of no response between Xuebijing injection and control groups in subgroup analyses of severe acute pancreatitis. Supplementary Material 6: Supplementary Table 1: the Preferred Reporting Items for Systematic Reviews and Meta-Analyses (PRISMA) checklist. Supplementary Material 7: Supplementary Table 2: meta-regression analyses. Supplementary Material 8: Supplementary Table 3: sensitivity analyses. Supplementary Material 9: Supplementary Table 4: publication bias. [file 6621368.f1.zip › 6621368.f1/Supplementary Table 4.docx]

| **Supplementary Table 4. Publication bias.** | | | | | |
| --- | --- | --- | --- | --- | --- |
| **Endpoints** | **Number of included studies** | **t** | **P** | **95%Confidence interval** | |
| ***Outcome*** | | | | | |
| **AP overall response** | 23 | 1.87 | 0.075 | -0.082 | 1.553 |
| SAP overall response | 17 | -0.99 | 0.341 | -1.031 | 0.381 |
| **AP complete response** | 23 | 1.04 | 0.309 | -0.641 | 1.931 |
| SAP complete response | 17 | -0.91 | 0.377 | -4.569 | 1.834 |
| **AP non-response** | 23 | -1.87 | 0.075 | -1.553 | 0.082 |
| SAP non-response | 17 | -2.98 | 0.010 | -48.693 | -7.908 |
| ***Laboratory indicators after treatment*** | | | | | |
| **IL-6 level** | 10 | -3.13 | 0.014 | -13.769 | -2.085 |
| **TNF-α level** | 12 | -3.68 | 0.004 | -25.171 | -6.178 |
| **AMS level** | 5 | -2.35 | 0.100 | -64.023 | 9.571 |
| **WBC** | 6 | -1.77 | 0.151 | -90.906 | 20.027 |
| **CRP level** | 5 | -2.27 | 0.108 | -40.460 | 6.793 |
| **hs-CRP level** | 4 | -0.71 | 0.553 | -126.312 | 90.688 |
| ***Recovery time of clinical symptoms and signs, laboratory indicators after treatment.*** | | | | | |
| **Abdominal pain** | 12 | 7.71 | 0.000 | 55.799 | 100.368 |
| **Abdominal distension** | 7 | 1.80 | 0.132 | -31.110 | 175.497 |
| **Gastrointestinal function** | 6 | 1.13 | 0.322 | -6.973 | 16.518 |
| **Body temperature** | 6 | 2.21 | 0.091 | -19.843 | 175.359 |
| **AMS level** | 5 | -0.07 | 0.952 | -22.887 | 21.961 |
| **WBC** | 8 | -1.88 | 0.109 | -10.656 | 1.397 |
| ***AP related complications*** | | | | | |
| **MODS** | 4 | 5.18 | 0.035 | 0.316 | 3.412 |
| **ARDS** | 3 | -2.33 | 0.258 | -46.728 | 32.224 |
| **Septicemia** | 3 | 0.83 | 0.560 | -27.656 | 31.507 |
| **Pancreatic pseudocyst** | 3 | 5.99 | 0.105 | -2.387 | 6.640 |
| **Abbreviations:** AP: acute pancreatitis; SAP: severe acute pancreatitis; IL-6: interleukin-6; TNF-α: tumor necrosis factor-α; AMS: serum amylase; WBC: white blood cell; CRP: C-reactive protein; hs-CRP: high sensitivity C-reactive protein; MODS: multiple organ dysfunction syndrome; ARDS: acute respiratory distress syndrome. | | | | | |
