# Supplementary figures and images for: Efficacy of Xuebijing Injection for Acute Pancreatitis: A Systematic Review and Meta-Analysis of Randomized Controlled Trials
Source: Evid Based Complement Alternat Med. 2021 Apr 26;2021:6621368. doi: 10.1155/2021/6621368 (PMC8214658; doi:10.1155/2021/6621368)

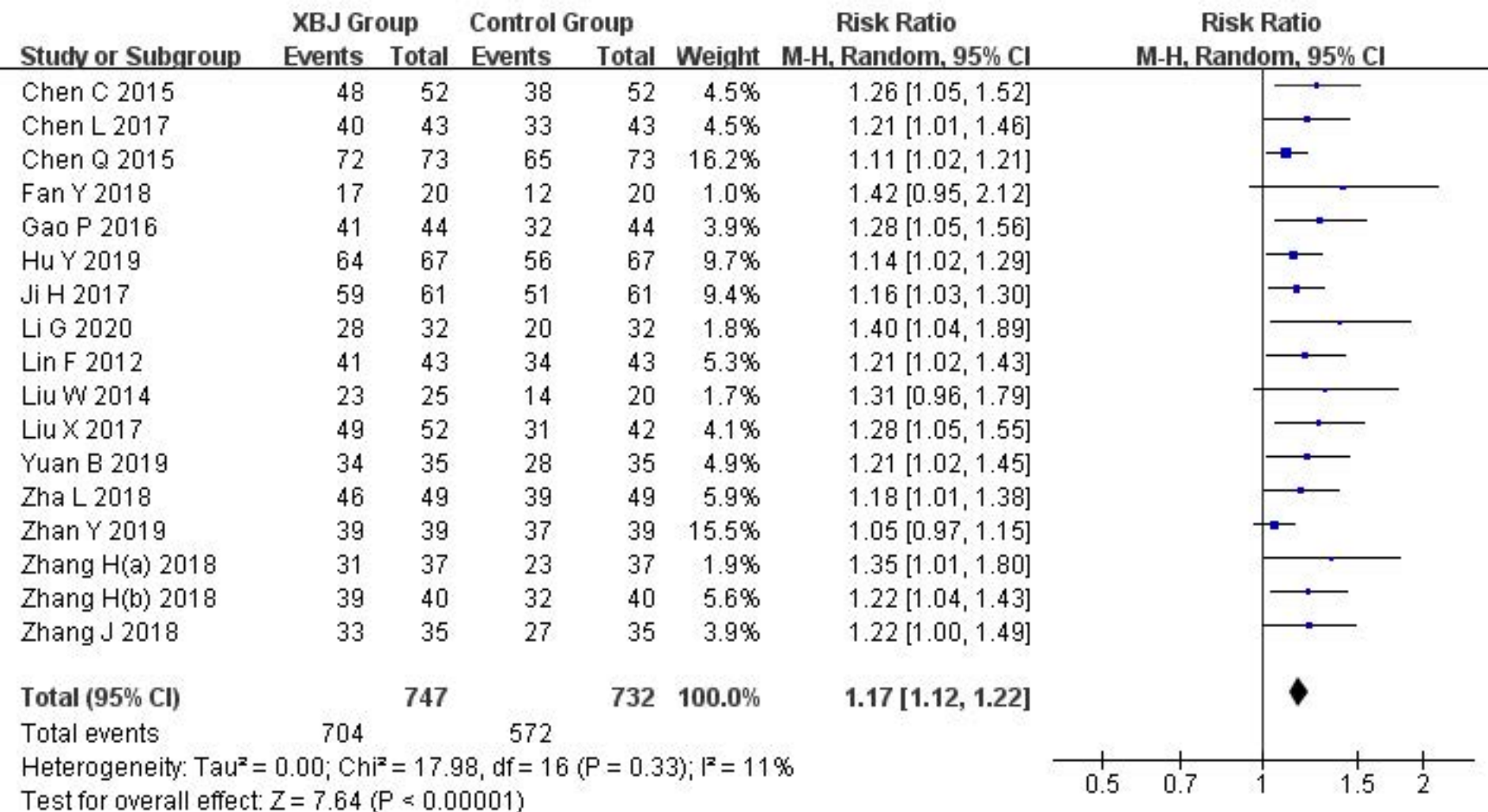

Supplement: Supplementary Materials — Supplementary Material 1: search strategy. Supplementary Material 2: Supplementary Figure 1: risk of bias assessment of potentially eligible papers. Supplementary Material 3: Supplementary Figure 2: comparison of overall response between Xuebijing injection and control groups in subgroup analyses of severe acute pancreatitis. Supplementary Material 4: Supplementary Figure 3: comparison of complete response between Xuebijing injection and control groups in subgroup analyses of severe acute pancreatitis. Supplementary Material 5: Supplementary Figure 4: comparison of no response between Xuebijing injection and control groups in subgroup analyses of severe acute pancreatitis. Supplementary Material 6: Supplementary Table 1: the Preferred Reporting Items for Systematic Reviews and Meta-Analyses (PRISMA) checklist. Supplementary Material 7: Supplementary Table 2: meta-regression analyses. Supplementary Material 8: Supplementary Table 3: sensitivity analyses. Supplementary Material 9: Supplementary Table 4: publication bias. [file 6621368.f1.zip › 6621368.f1/Supplementary Figure 2.pdf]

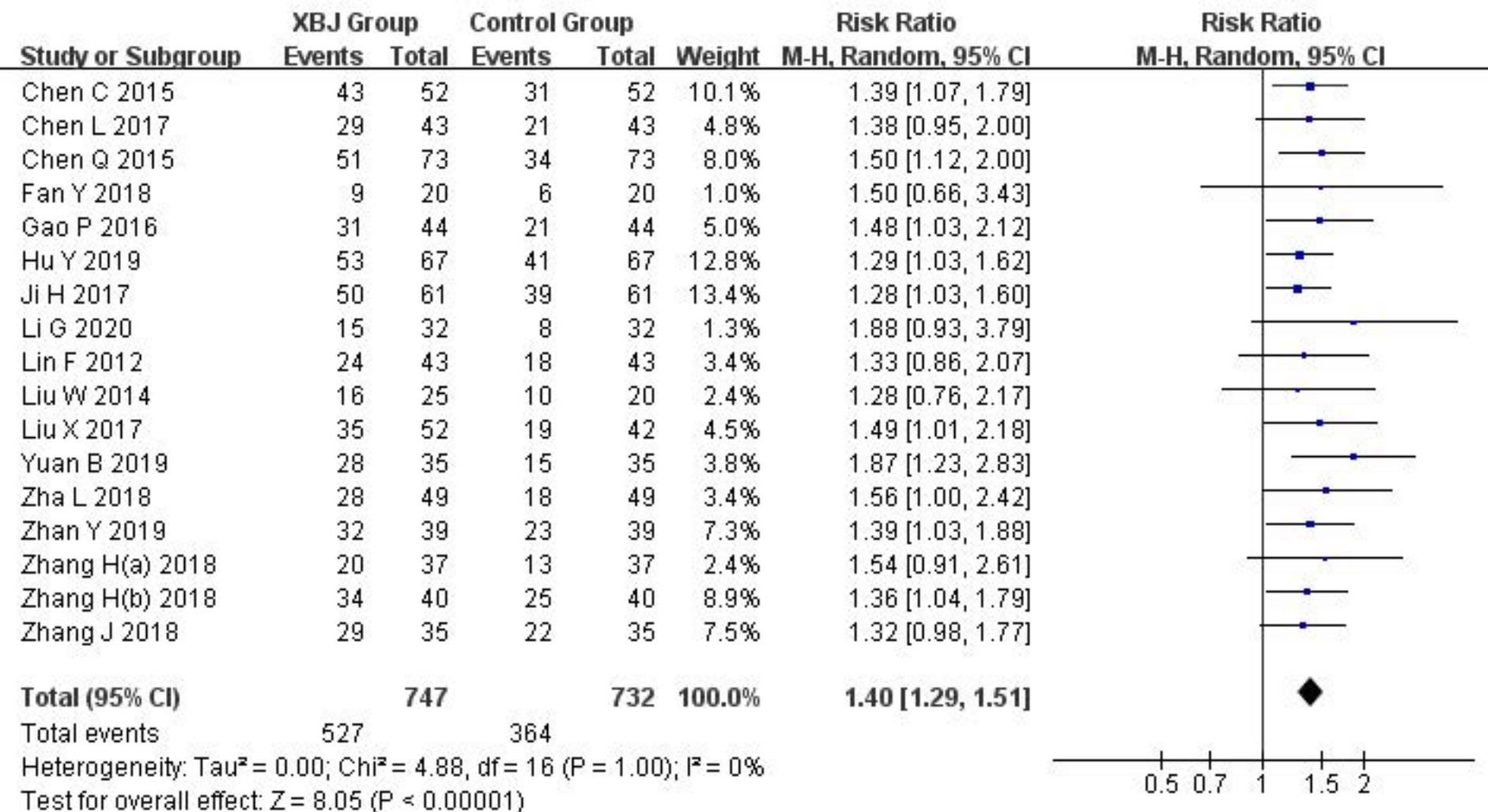

Supplement: Supplementary Materials — Supplementary Material 1: search strategy. Supplementary Material 2: Supplementary Figure 1: risk of bias assessment of potentially eligible papers. Supplementary Material 3: Supplementary Figure 2: comparison of overall response between Xuebijing injection and control groups in subgroup analyses of severe acute pancreatitis. Supplementary Material 4: Supplementary Figure 3: comparison of complete response between Xuebijing injection and control groups in subgroup analyses of severe acute pancreatitis. Supplementary Material 5: Supplementary Figure 4: comparison of no response between Xuebijing injection and control groups in subgroup analyses of severe acute pancreatitis. Supplementary Material 6: Supplementary Table 1: the Preferred Reporting Items for Systematic Reviews and Meta-Analyses (PRISMA) checklist. Supplementary Material 7: Supplementary Table 2: meta-regression analyses. Supplementary Material 8: Supplementary Table 3: sensitivity analyses. Supplementary Material 9: Supplementary Table 4: publication bias. [file 6621368.f1.zip › 6621368.f1/Supplementary Figure 3.pdf]

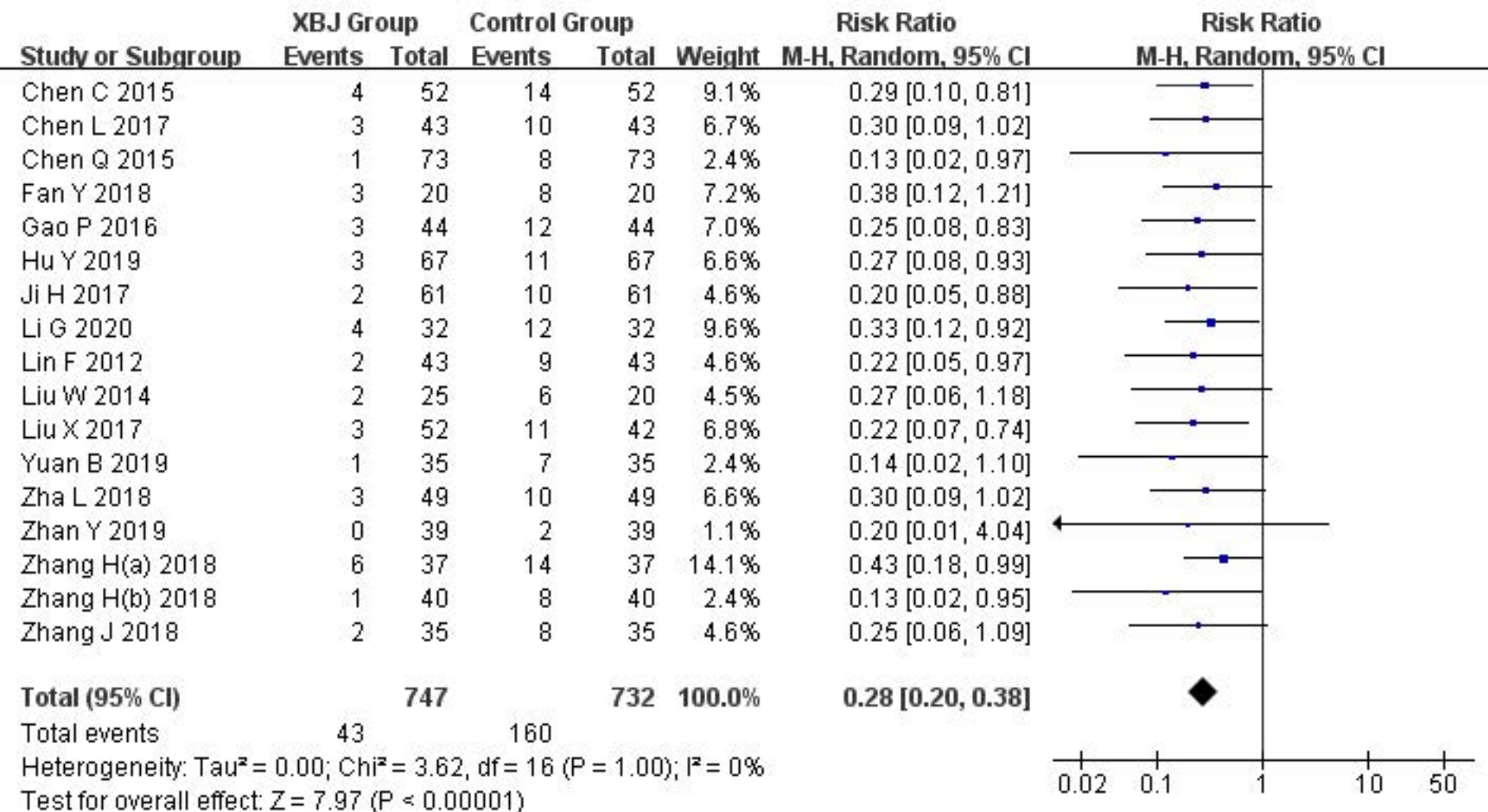

Supplement: Supplementary Materials — Supplementary Material 1: search strategy. Supplementary Material 2: Supplementary Figure 1: risk of bias assessment of potentially eligible papers. Supplementary Material 3: Supplementary Figure 2: comparison of overall response between Xuebijing injection and control groups in subgroup analyses of severe acute pancreatitis. Supplementary Material 4: Supplementary Figure 3: comparison of complete response between Xuebijing injection and control groups in subgroup analyses of severe acute pancreatitis. Supplementary Material 5: Supplementary Figure 4: comparison of no response between Xuebijing injection and control groups in subgroup analyses of severe acute pancreatitis. Supplementary Material 6: Supplementary Table 1: the Preferred Reporting Items for Systematic Reviews and Meta-Analyses (PRISMA) checklist. Supplementary Material 7: Supplementary Table 2: meta-regression analyses. Supplementary Material 8: Supplementary Table 3: sensitivity analyses. Supplementary Material 9: Supplementary Table 4: publication bias. [file 6621368.f1.zip › 6621368.f1/Supplementary Figure 4.pdf]
